# The Influence of Behavioral Sciences on Adherence to Physical Activity and Weight Loss in Overweight and Obese Patients: A Systematic Review of Randomized Controlled Trials

**DOI:** 10.3390/ijerph21050630

**Published:** 2024-05-16

**Authors:** Rafael Corrêa, Benjamin Miranda Tabak

**Affiliations:** School of Public Policy and Government, Getulio Vargas Foundation, SGAN 602 Módulos A,B,C, Asa Norte, Brasília 70830-020, Brazil; benjamin.tabak@fgv.br

**Keywords:** behavioral sciences, obesity, weight loss, physical activity

## Abstract

In recent years, weight gain and reduced physical activity in the general population have contributed to the development of obesity and other health problems; on the other hand, studies in behavioral sciences have been used to modify behaviors for a healthier life, so the objective of this study was to identify the evidence of interventions in behavioral sciences on adherence to physical activity and weight loss in obese patients. This systematic review study is based on a search of the electronic databases PubMed, Web of Science, Scopus, and Cochrane. Studies assessed the evidence from intervention studies that assessed the influence of intervention studies of behavioral sciences on public health. The articles were published between 2013 and 2023. The systematic search of the databases identified 2951 articles. The review analyzed 10 studies. Behavioral science interventions presented evidence through strategies such as multicomponent interventions, lottery and financial incentives, message framing, message framing with financial incentive and physical activity, and psychological satisfaction, demonstrating results in weight loss and maintenance and increased physical activity. This study presents scientific evidence through healthy behavior change methodologies, and future studies can explore these strategies in conjunction with public health technologies in the search for public–private partnerships to promote physical activity in adults.

## 1. Introduction

Obesity is a non-communicable disease (NCD) and has become a preventable public health problem, just like other chronic diseases that account for 70% of preventable deaths in the health area, affecting the population through cardiovascular risks, physical disabilities, high hospitalization rates, and increased healthcare costs, affecting people’s well-being and burdening the health system [1].

On the other hand, regular physical activity, understood as 150 min of moderate-intensity aerobic physical activity or 75 min of vigorous-intensity aerobic physical activity during the week, is recommended by the World Health Organization and world governments through practices for the adult population such as recreational or leisure physical activity, walking, cycling, domestic activities, sports games, and planned exercises, among others, which can be offered by health systems to the general population. Physical activity is a determining factor in changing individual and collective health behaviors, as it helps to improve cardiovascular fitness, reduce the risk of other chronic diseases, reduce weight, and improve quality of life [2,3,4,5]. 

However, although physical activity for overweight or obese people has shown advances in studies combined with other interventions, such as a gradual increase in physical activity [6], regular self-weighing [1], food choice and intake, and financial incentives and lotteries [7], maintaining weight loss in the long term has still been a challenge, as participants end up losing motivation due to the lack of continuous rewards for adhering to healthy behaviors [8]. Changing the architecture of choice presents advances in changing and maintaining health behaviors, but these must be conducted in a continuous program of physical activity to increase effectiveness [7,9]. 

Recognizing the important role of behavioral change strategies such as adherence to physical activity and weight loss in conjunction with cognitive and environmental change approaches for the prevention and treatment of non-communicable diseases, we highlight behavioral science approaches as innovative and low-cost and important for improving the quality of life of the population and optimizing the use of resources in the health area [5,6]. 

Behavioral sciences strategies have shown important results in physical activity and weight loss in an initial period; however, long-term maintenance of these healthy behaviors has been a challenge in the health field [7].

According to the theory of behavioral sciences, overweight or obese people tend to be more motivated by food-related activities than by activities without food reinforcement, which becomes another challenge for modifying the behavior of adherence to physical activity and weight loss. However, the weight of this reinforcement can be modified over time according to planned interventions or other social events or natural life events [8].

It is known that people try to change their sedentary behavior into regular physical activity through various attempts, often frustrated because it was not systematically planned. The behavioral sciences approach seeks to clarify this difficulty of behavioral change, based on some cognitive biases; one that applies to this described condition is the present bias, which can be described as a tendency to place greater value on present benefits compared to future costs, resulting in procrastination of healthy behaviors, and compensation mechanisms are developed to avoid this situation [2,9,10].

For this problem, behavioral sciences has presented evidence on the benefits of self-control and commitment devices through lotteries [11]; financial incentives to increase adherence to physical activity [12]; environmental modification strategies for changing eating behavior and increasing physical activity and weight control [13]; and regular self-weighing to prevent weight gain [14].

The systematic analysis of the effectiveness of the evidence presented by the behavioral science strategies listed in the previous paragraph is still a gap in the literature, thus increasing the need for and importance of conducting systematic review studies to identify behavioral science methods and strategies to improve the health behaviors of the general population. Therefore, this study seeks to analyze the influence of behavioral science interventions on obesity, weight loss, and adherence to physical activity in adults.

## 2. Materials and Methods

This systematic review used the PRISMA 2020 protocol [15] and was also registered with the Equator group through PROSPERO under the number CRD42023412377 (Table S1). 

The eligibility criteria for the studies in this systematic review was defined based on the following protocol: Randomized Controlled Trial studies with Behavioral Sciences interventions and Public Health outcomes, published between 2013 and 2023 in English, Portuguese, and Spanish. 

Indexed electronic databases such as Cochrane, PubMed, Web of Science, and Scopus were used to carry out the search, using the following strategy: (Public Health [MeSH Terms]) AND (behavioral economics (BE) [MeSH Terms]). Due to the number of studies in the initial search, the authors decided to divide them into priority themes, which for this study presented research involving behavioral sciences interventions in obesity, weight loss, and physical activity. Since behavioral sciences and public health are two intersectoral areas, the authors used a broad search strategy, obtaining a large number of articles for the sample (*n* = 85), in which case they opted to divide by related topics to facilitate analysis and discussion in specific areas. In this review, we present the results of intervention studies in Behavioral Sciences and the effect on physical activity and weight loss in obesity. 

Using the PICOS strategy described by the authors in the study protocol, we used the Rayyan software [16] to stratify and select the studies, which were selected through the identification and exclusion of duplicates, followed by the analysis of titles and abstracts according to the criteria described in the protocol. It should be noted that this analysis was carried out by three authors, being first carried out by two independent authors and supervised by a third author; however, the inconsistency in decisions was assisted by a third author, acting as a supporter in the decision-making process to obtain the study sample.

A similar process was used to extract and analyze the data. The extracted data were author, year, and country; study design; sample; instruments; study quality; and interventions and results, presented in tables and discussed in text form.

The process of analyzing the quality of the studies was carried out using the EBL checklist [17]. The instrument helps to assess the validity of studies using the following categories: sample, data collection, type of study, results, and overall validity. The subclasses were analyzed using the independent and overall percentage defined in the manual based on the following calculation, “Yes” ≥ 75% and “No/Unclear” ≤ 25% [17].

## 3. Results

**Figure 1:**
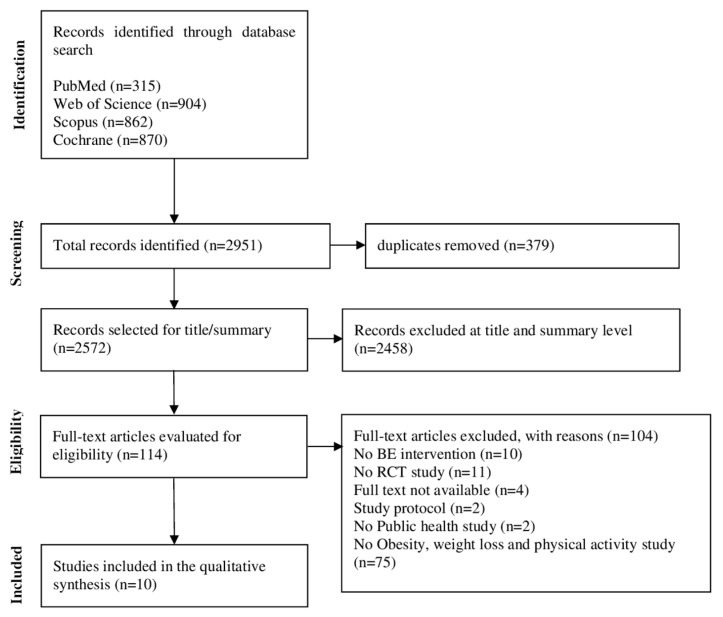
Study flowchart.

The authors identified 2952 studies using the strategy described above in the electronic databases; 379 articles were identified and removed as duplicates, and 2458 studies were analyzed and excluded using the titles and abstracts, according to the PICOS strategy described in the study protocol. Both the use of a broad search strategy in electronic databases as well as the use of RCT studies contributed to many studies being excluded from the analysis. A total of 114 studies were analyzed by reading the full text, and 75 studies were excluded because they did not meet the specific theme of the subdivision of this article, from which the authors obtained a sample of 10 studies. The systematic review flowchart is shown in Figure 1.

The final sample had an average validity of 60%, according to the EBL list [17], the results of which are shown in Table 1.

Regarding the type of studies, all the designs were randomized clinical trials (RCTs), defined as inclusion criteria. The participants involved in the studies were adults, aged between 18 and 80, with an average of 47 years. The majority of participants in the studies were women [7,12,21,22,23], followed by four studies that had a larger sample of men [1,2,24,25], and the sample sizes varied between 46 [25] and 344 participants [7].

Behavioral science interventions have been identified as multicomponent intervention [5,8,18]; lottery and financial incentives [2,19,20]; message framing [1,21]; message framing with financial incentives [22]; and physical activity and psychological satisfaction [23]. The duration of the interventions was between 30 min [22] and 24 months [7].

The results are presented in intervention blocks and in Table S2 to facilitate reading. The multicomponent intervention demonstrated a mean validity of the studies of 66%, showing results in weight reduction through reinforcement without food and physical activity [8], reducing the weight of participants in the study [5], and weight loss for participants in community psychiatric rehabilitation programs [18].

The lottery and financial incentives intervention had an overall average validity across studies of 56%, demonstrating evidence in weight loss and weight maintenance [19], increasing physical activity in the short term [2] and potentially supporting regular gym attendance in the long term [20]. 

The use of the message framing strategy showed an average validity across studies of 56%, demonstrating evidence in increasing participants’ physical activity [21] and being effective in regular self-weighing [1]. The use of message framing with financial incentives has shown that obese participants in stressful situations increase reinforcement for snack eating [22].

Finally, the intervention involving physical activity and psychological satisfaction showed that an increase in physical activity leads to a longer duration of pleasure and psychological satisfaction in the exercise and post-exercise periods, as well as in remembered pleasure [23].

## 4. Discussion

Behavioral science interventions have shown evidence through strategies such as multicomponent intervention, lottery and financial incentive, message framing, message framing with financial incentive, and physical activity and psychological satisfaction, demonstrating results in weight loss and maintenance and increased physical activity. 

Multicomponent interventions do not present a single, delimited strategic behavior, but instead the use of several strategies seeks to increase the chance of weight loss and increase participants’ physical activity. This process presents practical results; however, it makes it difficult to statistically analyze the data to attribute causality by comparing the quality of the evidence. This study demonstrates that in terms of cost, environmental interventions have a lower cost than financial incentives; however, a more detailed analysis with a comparative and effective cost assessment will be necessary for future studies delimiting the impact of each multicomponent intervention on weight loss and activity physical activity for the adult population and the influence on public health [5]. 

Individuals who are overweight or obese usually have physical limitations and high satisfaction with food-related incentives, and it is a necessary challenge to develop alternative strategies for adherence to the process of weight loss and physical activity. However, this process of change still needs to be clarified, whether it is the result of interventions or the process itself of losing weight and changing healthier habits [8]. 

The decision-making process of participants with overweight or obesity occurs based on the present and considering procrastination behavior in the search for immediate satisfaction. Studies in behavioral sciences encourage the identification of this pattern and change the logic by recommending increasing the proximate benefits (costs) of undesirable behaviors as a strategy for changing eating and physical activity behavior [19]. 

Physical activity is one of the factors for a sustained intervention for weight loss in patients in a multicomponent strategy, which is identified with high applicability in the health area compared to other strategies [24], followed by the use of commitment lotteries for overweight and obese patients [20].

In this sense, the use of lotteries is very useful considering the influence of weekly consequences, reducing the impact of procrastination. Research has used a similar strategy to support patient adherence to medication, as well as increased physical activity and weight loss [2,25].

For this type of intervention, the voluntary commitment of participants is an important factor in maintaining the desired behavior. A study identified people who are aware of the difficulty of self-control in the future and those who are not aware of this difficulty relating involvement in established deadlines and commitments. However, it is still unclear whether maintenance behavior is influenced by intrinsic or extrinsic motivation and the established reward [2].

It is worth highlighting the differentiation between the use of interventions with lotteries, commitment lotteries, and financial incentives. Commitment lotteries seek people who are already motivated to change, those who continue to maintain the desired behavior with an established commitment, and people who have not yet received the incentive. Conversely, the financial incentive aims to motivate people who are not in this condition of initial motivation to become motivated through the financial incentive [12]. In traditional lotteries, participants included in the study may receive the incentive within a specified period, regardless of involvement in the desired health behavior, which in this case would be weight loss or adherence to physical activity [12]. Therefore, it is important to analyze strategies and rewards in designing interventions based on the objective of the study, taking into account the power of motivation for the study sample [2]. 

Public and private health researchers, as well as professionals and public and private health managers, could make use of the technologies presented as part of an integrated care context through the use of prompts with deadlines established in agreement with patients to seek continuous modification of behaviors desirable in health [2,26].

Carrying out planned controlled studies in conjunction with electronic communication devices for monitoring the self-control of weight, the application of medication or glycemic control, monitoring of physical activity, cardiac effects by participants, and the research team is important for refining the evidence in health with a focus on weight loss and physical activity, as well as the establishment of partnerships with public and private entities that seek to ensure the continuity of the intervention study [19,27,28,29]. 

The knowledge developed by behavioral sciences in conjunction with physical exercise equipment and electronic technologies has already been improving the process of engaging in healthy behaviors through health apps (Healthy Wage, Pact, DietBet, Fitstudio, and Charity Miles) that encourage users to behaviors such as adhering to or increasing physical activity, losing and maintaining weight, and choosing and consuming healthy foods [21]. The possibility of using cards, which identified and tracked the total number of kilometers that participants covered during each exercise workout, converting them into “exercise credits” that could be applied to reimbursement of health insurance fees, charitable donations, and discounts on services, would facilitate the generalization of healthy behavior to other social contexts, developing more permanent habits in the general population [20].

The study has limitations, the first being the variety of interventions and results of the interventions analyzed, mainly related to multicomponent interventions, as it makes it difficult to analyze the influence of each intervention more accurately on the results of weight loss and physical activity. A second limitation can be attributed to using a broad search strategy in electronic databases, which may impact the number of studies in the systematic review. 

We highlight this review study’s use of a standardized protocol and assessment instrument as a strength. We also highlight the use of randomized controlled trial studies, which enable the causal relationship in the study evidence.

## 5. Conclusions

The behavioral sciences show satisfactory results with interventions around physical activity and weight loss, specifically through strategies such as multicomponent interventions, lottery, financial incentives, message framing, message framing with financial incentive and physical activity, and psychological satisfaction, demonstrating results in weight loss and maintenance and increased physical activity. With these results, managers, researchers, and the general population have scientific resources to make decisions in favor of more healthy development.

Further research could explore behavioral science interventions and public–private partnerships using health behavior change technologies and the impact on public health policies by improving adherence to physical activity for weight loss in obese patients. Future studies on interventions, including the evidence produced by behavioral sciences, could further optimize outcomes in prevention, treatment, and health promotion to improve physical activity in adults. 

## Figures and Tables

**Table 1:** Analysis of the EBL assessment checklist domains for the included study.

Studies	Validity (%)				General Validity of the Study (%)
	Population	Data Collection	Study Design	Result	
Buscemi et al., 2014 (USA) [8]	50	87	80	66	70
Glanz et al., 2019 (USA) [5]	50	75	80	33	59
Janssen et al., 2017 (USA) [18]	50	75	100	66	69
Piepmeier, Etnier and Fasczewski, 2018 (USA) [19]	33	87	100	66	8
Shaw et al., 2018 (USA) [5]	22	50	40	16	29
Snider et al., 2020 (USA) [20]	50	62	80	66	63
Takebayashi et al., 2022 (Japan) [1]	37	50	60	33	44
Van der Swaluw et al., 2018a (Netherlands) [2]	50	60	100	66	66
Van der Swaluw et al., 2018b (Netherlands) [21]	50	100	100	66	74
Zenko et al., 2016 (USA) [22]	37	87	80	33	59

## Data Availability

Protocol registration: Corrêa RS, Tabak BM, Systematic review protocol (CRD 42023412377). PROSPERO. Available at https://www.crd.york.ac.uk/PROSPEROFILES/412377_STRATEGY_20230329.pdf (accessed on 20 February 2024).

## Supplementary Materials

The following supporting information can be downloaded at: https://www.mdpi.com/article/10.3390/ijerph21050630/s1, Table S1: PRISMA 2020 Checklist. Table S2: Main findings and characteristics of studies on evidence from intervention studies.
