# Supplementary material for: The Influence of Behavioral Sciences on Adherence to Physical Activity and Weight Loss in Overweight and Obese Patients: A Systematic Review of Randomized Controlled Trials
Source: Int J Environ Res Public Health. 2024 May 16;21(5):630. doi: 10.3390/ijerph21050630 (PMC11121225; doi:10.3390/ijerph21050630)
Supplement: Supplementary file 1 [file ijerph-21-00630-s001.zip › ijerph-2976286-supplementary/Table S2. Main findings of the study.pdf]

Table S2. Main findings and characteristics of studies on evidence from intervention studies.

Author, year, country	Study design, sample	Instruments	Intervention	Study quality	Outcome
Buscemi et al., 2014 (USA)	Prospective randomized control trial. 202 overweight or obese participants adults (men (85) and women (117), >18 years, overage 51 years).	Demographics and anthropometrics.<br>The Activity Level Questionnaire–Eating Version (ALQ–EV).	The multicompenet intervention by meetings for weight loss and the development of a healthy lifestyle involved three components: cognitive-behavioral, diet prescription and physical activity prescription in two intervention groups a) group with food reinforcement and b) group without food reinforcement (18 months).<br>48 hours of group meetings.<br>Weekly (months 1 to 6) and twice a month (months 7 to 18).	70	The intervention demonstrated weight reduction through reinforcement without food and physical activity in overweight and obese patients.
Glanz et al., 2019 (USA)	Randomized controlled trial. 344 participants adults (men (97) and women (247), >18 years, overage 45 years).	Dietary behavior.<br>Physical activity.<br>Stages of change for weight loss.	The multicomponent intervention study used a web-based platform to track the participants' weight changes (24 months).<br>The intervention groups were: 1) control and	59	The intervention proved effective in reducing the weight of the study participants.

		Intrinsic motivation/treatment self-regulation.	standard care (weight measurement and wellness guidance); 2) daily lottery incentives (weight loss); 3) environmental strategy of dietary care and physical activity; and 4) combination of environmental strategy and lottery incentives.		
Janssen et al., 2017 (USA)	Randomized controlled trial. 291 participants adults (men (145) and women (146), >18 years, overage 45 years).	Weight loss, mean net weight loss.<br><br>Cost calculations, total contact time at one site by multiplying the person-hours per session by the expected number of sessions at one site.<br><br>Total staffing costs, multiplied the total time spent on the intervention by hourly compensation for staff.	The multicomponent intervention was developed in two phases: group weight loss meetings with message framing, individual weight loss visits, and group exercise classes (18 months).<br><br>The two phases differed only in terms of the frequency of meetings and visits.	69	The intervention resulted in weight loss for participants attending community psychiatric rehabilitation programs.
Piepmeier, Etnier & Fasczewski, 2018 (USA)	Randomized sample. 64 adults (men (30) and women (34), >18 years, overage 22 years).	Current Physical Activity Behaviour National Health Interview Survey.<br><br>Total of km cycled.<br><br>Polar Heart Rate monitor (F6 computer) with chest strap (T-31 Coded).	Message framing. Exposure to the 3-minute video "Exercise is Medicine™ - Keys to exercise - Exercise and your health" (30 min).<br><br>Three groups: 1) health (no additional information), 2) wealth (information that	68	The charity group intervention increased the participants' physical activity.<br><br>Women cycled further in the charity group, while men cycled further in the wealth group.

		Borg Rating of Perceived Exertion Scale.<br><br>Standard scale in behavioural economics research.	they would receive money per km cycled, and 3) charity (information that every 2 km they would convert the money earned to a charity)		
Shaw et al., 2018 (USA)	Randomized controlled trial. 188 participants adults,(>18 years).	Weights daily, Withings scale.	Intervention with lottery and financial incentives for weight loss and weight maintenance (6 months).<br><br>3 intervention groups: 1) lottery-based incentive, 2) financial incentive through direct payment, and 3) control through daily feedback without any incentive.	29	The study showed evidence of weight loss and weight maintenance in the participants.
Snider et al., 2020 (USA)	Randomized controlled trial. 48 participants adults (men (17) and women (31), >18 years, overage 40 years).	Three-Factor Eating Questionnaire (TFEQ-R-18).<br><br>Positive and Negative Affect Schedule (PANAS).<br><br>Ad libitum eating task.	Intervention with message framing and financial incentives (3 hours).<br><br>1) appetizer tasting test, 2) preference rating.<br><br>5 attempts to adjust the delay discount.<br><br>The product combinations for each of the three tasks were (a) money-now/money-later, (b) snack-now/money-later,	63	The intervention demonstrated that a stressful situation increases reinforcement for snack food in obese participants.

			and (c) snack-now/weight-loss-later.		
Takebayashi et al., 2022 (Japan)	Cluster randomized controlled trial. 122 participants adults (men (80) and women (42), >18 year, overage 35-39 years).	Survey of the three nudge groups:<br><br>Basic characteristics, number of subjects who had self-weighing habit after 6 months, changes in behavioral stage or mindset and weight maintenance.	Message framing intervention for regular self-weighing (6 months).<br><br>1 hour of workshops after being assigned to one of the nudge stimulus groups: 1) questionnaire group (attractive type nudges), 2) implementation intentions group (social type nudges) and 3) growth mindset group (timely type nudges), 4) reference group (no stimuli).	44	All the interventions were effective in regular self-weighing, but the growth mindset intervention was more effective.
van der Swaluw et al., 2018a (Netherland)	Cluster randomized trial. 163 overweight participants adults (men (113) and women (50), >18 years, overage 48 years).	Goal attainment (week gym attendance ≥ 2)	Lottery with finantial incentives intervention to increase physical activity in overweight adults (6 months).<br><br>1) Weekly short-term lotteries (13 weeks - 100 euros); 2) Combination of short-term and long-term lotteries (26 weeks - 5,400 euros); and 3) Control group with no lotteries.	66	The intervention proved effective in increasing physical activity in the short term.
Van der Swaluw et al., 2018b (Netherlands)	Cluster randomized trial. 163 overweight participants adults	Goal-attainment (week-gym attendance ≥ 2)	Lottery with finantial incentives intervention to increase physical activity in	74	Commitment lotteries can support regular gym attendance over the long term (up to 52 weeks).

	(men (113) and women (50), >18 years, overage 48 years).	Scales to measure weight (KERNTM; 0.1% precision).	overweight adults (6 months).<br><br>1) Weekly short-term lotteries (13 weeks - 100 euros); 2) Combination of short-term and long-term lotteries (26 weeks - 5,400 euros); and 3) Control group with no lotteries.		
Zenko et al., 2016 (USA)	Randomized controlled trial. 46 adutls (men (31) and women (15), >18 years, overage 28 years).	Feeling Scale.<br><br>Visual analog scale (VAS).<br><br>Empirical Valence Scale (EVS).<br><br>Physical Activity Enjoyment Scale (PACES).<br><br>Rating of Perceived Exertion (RPE).<br><br>Incidental and Planned Exercise Questionnaire (IPEQ-WA).	The physical exercise and psychological satisfaction intervention took place in laboratory visits: (a) maximal exercise test, (b) experimental exercise and (c) evaluation of the results (1 week).<br><br>2 groups of recumbent cycling tasks of increasing intensity (a) or decreasing intensity (b).	59	The intervention with the increasing intensity group showed a greater duration of pleasure and satisfaction in the post-exercise period as well as in the remembered pleasure.
